# Genetic and Adverse Health Outcome Associations with Treatment Resistant Hypertension in GenHAT

**DOI:** 10.1155/2013/578578

**Published:** 2013-10-31

**Authors:** Amy I. Lynch, Marguerite R. Irvin, Barry R. Davis, Charles E. Ford, John H. Eckfeldt, Donna K. Arnett

**Affiliations:** ^1^Department of Epidemiology, University of Alabama at Birmingham, RPHB 220E, 1530 3rd Avenue South, Birmingham, AL 55294-0022, USA; ^2^School of Public Health, The University of Texas Health Science Center at Houston, Houston, TX 20186, USA; ^3^Department of Laboratory Medicine and Pathology, University of Minnesota, Mayo Mail Code 609, 420 Delaware Street SE, Minneapolis, MN 55455, USA

## Abstract

Treatment resistant hypertension (TRH) is defined as uncontrolled hypertension (HTN) despite the use of ≥3 antihypertensive medication classes or controlled HTN while treated with ≥4 antihypertensive medication classes. Risk factors for TRH include increasing age, diminished kidney function, higher body mass index, diabetes, and African American (AA) race. Importantly, previous studies suggest a genetic role in TRH, although the genetics of TRH are largely understudied. With 2203 treatment resistant cases and 2354 treatment responsive controls (36% AA) from the Genetics of Hypertension Associated Treatment Study (GenHAT), we assessed the association of 78 candidate gene polymorphisms with TRH status using logistic regression. After stratifying by race and adjusting for potential confounders, there were 2 genetic variants in the AGT gene (rs699, rs5051) statistically significantly associated with TRH among white participants. The Met allele of rs699 and the G allele of rs5051 were positively associated with TRH: OR = 1.27 (1.12–1.44), *P* = 0.0001, and OR = 1.36 (1.20–1.53), *P* < 0.0001, respectively. There was no similar association among AA participants (race interaction *P* = 0.0004 for rs699 and *P* = 0.0001 for rs5051). This research contributes to our understanding of the genetic basis of TRH, and further genetic studies of TRH may help reach the goal of better clinical outcomes for hypertensive patients.

## 1. Introduction

Multidrug resistance to blood pressure (BP) lowering treatments is a growing problem in the USA [1]. In a recent report based on the National Health and Nutrition Examination Surveys (NHANES) collected in 1998–1994, 1999–2004, and 2005–2008, the percentage of treated uncontrolled patients on monotherapy fell, whereas the percentage on at least 3 medications increased over time [1]. Among those most severely affected are persons with treatment resistant hypertension (TRH) who have uncontrolled hypertension (HTN) on ≥3 antihypertensive medication classes or require ≥4 antihypertensive medications to reach their BP goals [2]. Recently, multiple studies have reported that the prevalence of TRH is 10–20% among those with HTN. However, some limitations of these estimates in the context of epidemiological studies are noted (e.g., potential misclassification due to BP measuring technique, nonadherence to medication, inadequate dosing, white-coat hypertension, and/or secondary causes of HTN) [3, 4]. Most concerning is the fact that TRH is consistently associated with increased cardiovascular disease (CVD) morbidity and mortality compared to more easily controlled HTN [3, 4].

Demonstrated risk factors for TRH include increasing age, diminished kidney function, higher body mass index (BMI), diabetes mellitus (DM), and African American race (e.g., African Americans are approximately twice as likely to be affected [1, 5]). However, these risk factors only partially account for the incidence of TRH, suggesting that other factors, including genetic factors, may increase risk [6]. A handful of small candidate gene studies suggest a genetic component for TRH, although the genetics of TRH are incompletely understood and largely understudied [7–9]. Therefore, we assessed genetic associations with TRH among African American and white participants in the Genetics of Hypertension Associated Treatment (GenHAT) study using 78 candidate genetic polymorphisms implicated in the development of hypertension and CVD. GenHAT leverages rich clinical data collected by the Antihypertensive and Lipid lowering Treatment to Prevent Heart Attack Trial (ALLHAT) [10, 11]. Strengths of ALLHAT/GenHAT for the current study of TRH are that drug utilization and dose optimization are well documented, in addition to adherence data, resulting in less potential misclassification of TRH. Ultimately, better understanding of genetic risk may improve clinical care for TRH and prevent associated CVD morbidity and mortality.

## 2. Materials and Methods

### 2.1. Study Design

These data were derived from the GenHAT study (*N* = 39,114), an ancillary study to the Antihypertensive and Lipid-Lowering Treatment to Prevent Heart Attack Trial (ALLHAT). ALLHAT included 42,418 hypertensive participants aged 55 years and older (46% women and 35% African American) who had one or more known CVD risk factors in addition to hypertension. ALLHAT was a randomized, double-blind, multicenter (*n* = 623 centers) clinical trial which tested whether the incidence of fatal CHD and nonfatal MI was lower with three antihypertensive drug classes (i.e., a calcium channel blocker, an angiotensin converting enzyme (ACE) inhibitor, and an alpha-adrenergic blocker), compared to treatment using a thiazide-type diuretic. Participants were randomized to treatment in a ratio of 1 : 1 : 1 : 1.7 for amlodipine, lisinopril, doxazosin, and chlorthalidone, respectively, to optimize statistical power for the three primary comparisons of ALLHAT [12]. The treatment goal was to achieve BP less than 140/90 mmHg by (1) titrating doses of the assigned study drug, and, if still necessary to control BP and (2) adding an open label drug (atenolol, reserpine, or clonidine as step 2 and hydralazine as step 3). To understand gene-treatment interactions on BP and CVD outcomes, GenHAT genotyped variants in several HTN- and CVD-related genes among 39,114 ALLHAT participants with available DNA. Complete descriptions of the rationale and design of ALLHAT and GenHAT have been previously published [12, 13]. This research was approved by local Institutional Review Boards. Genetic data were anonymized.

### 2.2. Study Population

The extreme phenotype design has been shown to be an efficient and powerful approach for genetic discovery in complex disease [14, 15]. Therefore, the cases and controls selected for the current effort represent extremes of the distribution of number of antihypertensive medications required to control HTN in GenHAT. Specifically, treatment resistant hypertension (TRH) cases were defined as all participants taking three antihypertensive medications at the ALLHAT year-3 follow-up visit who had a systolic BP (SBP) >140 mmHg or diastolic BP (DBP) >90 mmHg or taking greater than three antihypertensive medications regardless of BP at that visit. Easily-treated controls are defined as those taking only the randomized antihypertensive drug at year-3 and having a year-3 SBP≤125 and DBP≤85. To reduce the chance that lack of adherence to prescribed treatment, rather than true treatment resistance, accounts for the observed difficulty in controlling BP, we included only those participants who reported taking 80% or more of randomized drug at more than 50% of clinic visits. Using the classification scheme above, among the African-American participants with genotype data (*n* = 13,544), 6.9% (*n* = 929) were classified as TRH cases and 5.3% (*n* = 719) were classified as easily-treated controls. Among the white participants with genotype data (23,657), 5.4% (*n* = 1,274) were TRH cases and 6.9% (*n* = 1,635) were controls. 

### 2.3. Outcome Ascertainment and Blood Pressure Measurement

The BP values used to classify participants as TRH cases or controls were measured at the year-3 postrandomization clinic visit. Trained observers using standardized techniques measured SBP, defined as the first Korotkoff sound (1st phase), and DBP, defined as the reading at the last Korotkoff sound (5th phase). The measurements were taken only after the participant had been seated quietly for at least 5 minutes with feet flat on the floor and the arm at or as close to the level of the heart as possible. The cuff was deflated at a rate of 2 mm/sec until 10 mm Hg below the level of the diastolic reading was reached. Two measurements were taken, with at least a 30-second interval between the measurements, and recorded to the nearest even number. All BP measurements were calculated as the average of these two readings [16].

Outcomes compared between TRH cases and controls were fatal CHD and nonfatal MI (hereafter referred to as “CHD”), stroke, heart failure (fatal, requiring hospitalization, or treated in an outpatient setting), end-stage renal disease (ESRD), all-cause mortality, combined CHD (includes CHD + coronary revascularization + hospitalized angina), and combined CVD (includes combined CHD + stroke + treated angina without hospitalization + heart failure [fatal, hospitalized, or treated in an outpatient setting] + peripheral arterial disease [in hospital or outpatient revascularization]), with a mean follow-up of 4.9 years. Outcomes were reported by clinical investigators, and documentation (hospital discharge summary, death certificate) was submitted for any outcome involving hospitalization or death. Deaths occurring among participants lost to follow-up were also identified using national databases. Outcomes were not mutually exclusive; for example, the primary outcome (CHD) was included in the “combined CHD” outcome, and the combined CHD outcome (as well as stroke and CHF) was included in the “combined CVD” outcome, each outcome being more inclusive than the previous one. A detailed description of outcome ascertainment for ALLHAT has been previously published [12, 17, 18].

### 2.4. Genotyping Methods

DNA was isolated on FTA paper (Fitzco Inc, Maple Plain, MN, USA) from blood samples. Genotyping was performed for 78 candidate polymorphisms using amplified DNA products of a multiplex PCRs and detected using an immobilized probe research assay for multiple candidate markers (“Roche strip,” Roche Molecular Systems, Pleasanton, CA, USA) as described previously [19]. These variants were selected for inclusion by Roche because there was evidence that the biochemical pathways of these genes were implicated in the development and progression of CVD. See Table 1 for a complete list of genetic variants tested.

### 2.5. Statistical Analysis

To test for differences in baseline characteristics between TRH cases and controls in Table 2, ANOVA was used for continuous variables and chi-square tests were used for categorical variables. For Table 3, logistic regression was used to test for differences in clinical outcomes between TRH cases and controls in both unadjusted models and after adjusting for age, sex, randomized treatment group, baseline BMI, HDL, estimated glomerular filtration rate, SBP and DBP, smoking status, diabetes status, and history of left ventricular hypertrophy as detected by ECG. For Table 4, logistic regression was used to test for genetic associations with TRH, after adjusting for the same set of covariates. An additive genetic model was used for each variant, with common homozygotes coded 0, heterozygotes coded 1, and rare homozygotes coded 2; therefore, the ORs in Table 4 represent the odds of being in the TRH case group versus the control group for each copy of the minor allele. To reduce the chance that genetic admixture would lead to spurious associations, we analyzed a total of 78 variants (Table 1) in a self-identified race-specific manner. Because we performed 156 tests of genetic associations (78 × 2), a strict Bonferroni correction for multiple tests would require a *P* value of less than (0.05/156) = 0.00032 to be considered statistically significant. All statistical analyses were performed using STATA version 10.1 (STATA Corporation, College Station, TX, USA).

## 3. Results and Discussion

### 3.1. Results

Baseline characteristics and follow-up BP measures for TRH cases and controls are shown in Table 2. Generally, TRH cases were more likely to be male, diabetic (Type 2), heavier, and taking BP medication at baseline. Cases also had higher baseline fasting glucose and BP and lower HDL cholesterol and estimated GFR, when compared to controls. There were also differences in case-control status depending on randomization group: those in the chlorthalidone and amlodipine groups were more likely to be controls, whereas those in the lisinopril and doxazosin groups were more likely to be cases.

Table 3 shows the association of the ALLHAT clinical outcomes with TRH. After adjusting for potential confounders, TRH was positively associated with CHD (OR = 3.44, *P* < 0.001), heart failure (OR = 2.69, *P* < 0.001), all-cause death (OR = 1.66, *P* = 0.01), combined CHD (OR = 2.84, *P* < 0.001), and combined CVD (OR = 2.11, *P* < 0.001), but not stroke, among the African-American participants. There were also 28 incident ESRD events among the TRH group, and none among the control group for the African Americans. Among the white participants, TRH was positively associated with CHD (OR = 2.40, *P* < 0.001), stroke (OR = 2.19, *P* = 0.003), heart failure (OR = 3.98, *P* < 0.001), ESRD (OR = 3.89, *P* = 0.02), combined CHD (OR = 2.22, *P* < 0.001), and combined CVD (OR = 2.42, *P* < 0.001).

Among the 78 genetic variants tested, there were no statistically significant genetic associations with TRH for the African-American participants after correcting for multiple comparisons. There were 2 genetic variants, both located in the angiotensinogen (AGT) gene, showing significant associations among white participants after adjusting for potential confounders and correcting for multiple comparisons: AGTM235T rs699 and AGT-6 rs5051 (shown in Table 4). The Met allele of AGTM235T rs699 and the G allele of AGT-6 rs5051 were positively associated with TRH: OR = 1.27 (1.12–1.44), *P* = 0.0001, and OR = 1.36 (1.20–1.53), *P* < 0.0001, respectively. There was no similar association among African-American participants, although it should be noted that the Met (M) allele of AGTM235T rs699 and the G allele of AGT-6 rs5051 are far less frequent among African-American than white participants. There was evidence of a significant interaction by race in the association of both AGTM235T rs699 and AGT-6 rs5051 with TRH when combining the data from whites and African-Americans (interaction *P* = 0.0004 for rs699 and *P* = 0.0001 for rs5051).

There were 3 genetic variants that showed suggestive evidence (*P* < 0.05) of association with TRH among both the African-American and white participant groups (see Table 5). For methylenetetrahydrofolate reductase (NAD(P)H) (MTHFR) C667T rs1801133, the minor allele (T) was associated with lower odds of TRH compared to the common allele (C) for both African Americans (OR = 0.67 [0.52–0.86], *P* = 0.002) and whites (OR = 0.88 [0.77–0.99], *P* = 0.04). For the matrix metalloproteinase-3 (MMP3) 5A/6A rs3025058 variant, however, the association with TRH differs between the races. For African Americans, the minor allele was protective (OR = 0.75 [0.57–0.98], *P* = 0.04), whereas for whites the minor allele confers higher odds of TRH (OR = 1.14 [1.01–1.29], *P* = 0.03). For the tumor necrosis factor (TNF) rs361525 variant, the minor allele (A) is positively associated with TRH among both African-Americans (OR = 1.87 [1.13–3.11], *P* = 0.02) and white participants (OR = 1.44 [1.10–1.90], *P* = 0.009). It should be noted that the frequency of the minor alleles for rs1801133, rs3025058, and rs361525 was much more common among white participants compared to African Americans (see Table 5 for genotype frequencies). 

### 3.2. Discussion

The follow-up BP data (Table 2) shows that from baseline to years 1, 2, and 3, the control group was able to substantially reduce their BP, whereas the TRH cases did not see marked improvement in BP control, despite the additional antihypertensive medications prescribed. There is also strong evidence among the GenHAT participants that TRH is associated with a higher burden of cardiovascular disease, renal disease, and death (Table 3) compared to those with more easily treated HTN. Other studies have considered risk for incident CVD events associated with TRH in comparison to more easily treated HTN, although their results cannot be directly compared to data from the current study due to differences in the comparison group. Still they draw consistent conclusions that persons with TRH are at higher risk for CVD outcomes. Previously published ALLHAT research identified factors predicting poorer BP control at the 3 year visit, including increased age, baseline SBP, and BMI, as well as African-American race, diabetes (type 2), and female gender [16]. Analyses of the full ALLHAT cohort for CVD outcomes in TRH are currently underway.

Two AGT genetic variants (rs699 and rs5051) were statistically significantly associated with TRH among the white participants, but no such associations were found for the African-American participants. This finding is in the opposite direction of a recent smaller study (70 TRH cases among Brazilian patients) showing that carriers of the *AGT *M235T Thr allele were at increased risk for resistant hypertension, particularly those participants older than 50 years, concluding that the rs699 (*AGT* M235T) Thr allele was an independent risk factor for resistant hypertension. Although they did not report the racial make-up of the participant groups, the allele frequencies reported for their population were more similar to the GenHAT white group than the African-America group [20]. Although *AGT* genetic variants are among the most studied in the context of hypertension and cardiovascular disease, and many studies have reported significant associations, the directions of the associations are not consistent [10, 11, 21–26]. There is also previous evidence that hyperaldosteronism causes treatment resistant hypertension [27, 28], which is important since both aldosterone and AGT are components of the renin-angiotensin aldosterone system (RAAS), which has a strong role in the regulation of BP.

Although not statistically significant, it is of interest that 3 genetic variants (MTHFR C667T rs1801133, MMP3 5A/6A rs3025058, and TNF rs361525) showed suggestive evidence of association with TRH among both the African-American and white populations. To varying degrees, all three of these genes have been previously studied in the context of hypertension. The matrix metalloproteinase (MMP) enzymes are inflammatory mediators that may contribute to hypertension, and though many previous studies which have shown an association with the C667T variant have found the minor T allele to be high risk, our data show the C allele to be more common among the TRH cases than the controls among both white and African-American participants. Previous studies have assessed the effect of MMP genes on hypertension and its sequelae, with an over expression of MMP found in hypertension [29–32]. We observed an inconsistent association in our data for the MMP3 rs3025058 between the white and African-American participants, although the minor allele was far less common among African-American compared to white participants. Tumor necrosis factor (TNF) is a cytokine involved in a wide variety of functions including cytolysis of tumor cell lines and the induction of cachexia, pyrogenicity, cell proliferation, and cell differentiation in some situations [33]. Although TNF genes have not been widely studied as predictors of hypertension, several studies have found that TNF interacts with the RAAS system in the regulation of BP [34–38].

It is not surprising that both AGT rs699 and rs5051 show similar associations, as the AGT −6 and M235T variants are in tight linkage disequilibrium (LD) on chromosome 1. Using SNAP (SNP Annotation and Proxy Search) [39], the LD between rs699 and rs5051 is *r* ^2^ = 0.964 and *r* ^2^ = 0.908 in the HapMap CEU population panel (Utah residents with Northern and Western European ancestry) and the HapMap YRI population panel (Yoruba residents of Ibadan, Nigeria), respectively. Both of these variants were in Hardy-Weinberg equilibrium (HWE) for our study population when tested in a race-specific manner (rs699 HWE *P* = 0.33 and *P* = 0.05 for whites and African Americans, resp., and rs5051 HWE *P* = 0.38 and *P* = 0.06 for whites and African Americans, resp.).

The strengths of this study include the relatively large number of TRH cases available in the ALLHAT/GenHAT study, including large numbers of African Americans with TRH. In addition, in the context of a randomized trial, we were better able to exclude pseudoresistant HTN cases characterized by poor BP measuring technique, nonadherence to medication, and/or inadequate dosing than a population-based study. In doing so, we observe a lower TRH prevalence in GenHAT versus other previous reports. Limitations of this case-control study include the loss of participants to death and other events, who might otherwise have been classified as TRH, as well as the inability to control for unknown differences between groups. In addition, ALLHAT recruited only patients aged 55 years and older, which does not allow for assessing the genetic effects on TRH among a younger population. Because a stronger genetic propensity may be present among those who manifest TRH at an early age, future prospective studies of the genetics of TRH would be well-advised to include a broad age range, including a younger hypertensive population. 

## 4. Conclusions

Improving prevention and treatment of TRH will be enhanced with a thorough knowledge of the genetic architecture of TRH. Understanding the underlying etiology and progression of this disorder can potentially generate new ideas for treatment. This research contributes to our understanding of the genetic basis of TRH, and further studies of TRH may help us reach the goal of better clinical outcomes for hypertensive patients. 

## Figures and Tables

**Table 1:** Genetic variants analyzed.

SNP rs number	Gene name	Gene symbol	Alleles
1799752	Angiotensin I-converting enzyme	ACE	Insertion/deletion
4363	Angiotensin I-converting enzyme	ACE	A/G
4291	Angiotensin I-converting enzyme	ACE	A/T
4343	Angiotensin I-converting enzyme	ACE	A/G
4961	Alpha adducin	ADD1	G/T
1042713	Beta-2-adrenergic receptor	ADRB2	G/A
1042714	Beta-2-adrenergic receptor	ADRB2	C/G
1800888	Beta-2-adrenergic receptor	ADRB2	C/T
5050	Angiotensin I; angiotensinogen	AGT	A/C
5051	Angiotensin I; angiotensinogen	AGT	A/G
699	Angiotensin I; angiotensinogen	AGT	C (thr)/T (met)
5186	Angiotensin receptor I	AGTR1	A/C
1492078	Angiotensin receptor I	AGTR1	A/G
275653	Angiotensin receptor I	AGTR1	T/C
676210	Apolipoprotein B	APOB	C/T
1042031	Apolipoprotein B	APOB	G/A
5742905	Cystathionine-beta-synthase	CBS	Ile/thr
1799963	Coagulation factor II	F2	G/A
6025	Coagulation factor V	F5	G/A
6046	Coagulation factor VII	F7	G/A
5742910	Coagulation factor VII	F7	Insertion/deletion
7981123	Coagulation factor VII	F7	G/T
762637	Coagulation factor VII	F7	G/A
1801020	Coagulation factor XII	F12	C/T
5982	Coagulation factor Xll1, Al subunit	F13	C/T
5985	Coagulation factor Xll1, Al subunit	F13	C/T
1800790	Fibrinogen, B beta polypeptide	FGB	G/A
7121	Guanine nucleotide-binding protein, alpha-stimulating polypeptide 1	GNAS	T/C
5443	Guanine nucleotide-binding protein, BETA-3	GNB3	C/T
6065	Platelet glycoprotein Ib (alpha polypeptide)	GP1BA	C/T
4069688	Platelet glycoprotein Ib (alpha polypeptide)	GP1BA	G/T
2243093	Platelet glycoprotein Ib (alpha polypeptide)	GP1BA	T/C
1024323	G protein-dependent receptor kinase 4	GRK4/GPRK2L	C/T
1129292	G protein-dependent receptor kinase 4	GRK4/GPRK2L	C/T
2960306	G protein-dependent receptor kinase 4	GRK4/GPRK2L	G/T
1799969	Intercellular adhesion molecule 1	ICAM 1	G/A
1062535	Integrin, alpha-2	ITGA2	G/A
5918	Integrin, beta-3	ITGB3	T/C
328	Lipoprotein lipase	LPL	C/G
1041981	Lymphotoxin-alpha	LTA	C/A
1799750	Matrix metalloproteinase 1	MMP1	1G/2G
243865	Matrix metalloproteinase 2	MMP2	C/T
3025058	Matrix metalloproteinase 3	MMP3	5A/6A
11568818	Matrix metalloproteinase 7	MMP7	A/G
11568819	Matrix metalloproteinase 7	MMP7	C/T
2664538	Matrix metalloproteinase 9	MMP9	A/G
2274756	Matrix metalloproteinase 9	MMP9	G/A
2276109	Matrix metalloproteinase 12	MMP12	A/G
652438	Matrix metalloproteinase 12	MMP12	A/G
1801131	Methylenetetrahydrofolate reductase	MTHFR	A/C
1801133	Methylenetetrahydrofolate reductase	MTHFR	C/T
1799983	Nitric oxide synthase 3	NOS3	G/T
3918226	Nitric oxide synthase 3	NOS3	C/T
1800779	Nitric oxide synthase 3	NOS3	A/G
5065	Natriuretic peptide precursor A	NPPA	T/C
5063	Natriuretic peptide precursor A	NPPA	G/A
7242	Plasminogen activator inhibitor 1	PAI1/SERPINE1	T/G
1799768	Plasminogen activator inhibitor 1	PAI1/SERPINE1	5G/4G
27727	Phosphodiesterase 4D	PDE4D	A/G
40512	Phosphodiesterase 4D	PDE4D	A/G
10074908	Phosphodiesterase 4D	PDE4D	A/G
702553	Phosphodiesterase 4D	PDE4D	T/A
12188950	Phosphodiesterase 4D	PDE4D	G/A
6450512	Phosphodiesterase 4D	PDE4D	T/C
153031	Phosphodiesterase 4D	PDE4D	A/G
27653	Phosphodiesterase 4D	PDE4D	C/A
456009	Phosphodiesterase 4D	PDE4D	C/T
705379	Paraoxonase I	PON1	C/T
6681776	Renin	REN	G/A
2368564	Renin	REN	C/T
5742912	Sodium channel, nonvoltage-gated 1, alpha subunit	SCNNIA	T/C
2228576	Sodium channel, nonvoltage-gated 1, alpha subunit	SCNNIA	G/A
5361	Selectin E	SELE	A/C
5355	Selectin E	SELE	C/T
361525	Tumor necrosis factor	TFN	G/A
673	Tumor necrosis factor	TFN	G/A
1800629	Tumor necrosis factor	TFN	G/A
1800750	Tumor necrosis factor	TFN	G/A

**Table 2:** Baseline characteristics for African-American and white treatment resistant hypertension cases and controls.

Characteristic	African-American participants			White participants		
	TRH cases	Controls	*P* value*	TRH cases	Controls	*P* value*
Sample size, *n*	929	719	—	1274	1635	—
Age (y), mean (SD)	65.8 (7.1)	65.7 (7.4)	0.92	67.4 (7.2)	66.6 (7.5)	0.003
Women, *n* (%)	440 (47.4)	391 (54.4)	0.005	440 (34.5)	712 (43.6)	<0.001
On antihypertensive treatment at baseline, *n* (%)	892 (96.0)	618 (86.0)	<0.001	1221 (95.8)	1448 (88.6)	<0.001
Randomized treatment group:						
Chlorthalidone**, *n* (col%)	253 (27.2)	366 (50.9)	<0.001	420 (33.0)	695 (42.5)	<0.001
Amlodipine, *n* (col%)	166 (17.9)	161 (22.4)		236 (18.5)	349 (21.4)	
Lisinopril, *n* (col%)	301 (32.4)	118 (16.4)		383 (30.1)	429 (26.2)	
Doxazosin, *n* (col%)	209 (22.5)	74 (10.3)		235 (18.5)	162 (9.9)	
Baseline blood pressure:						
All participants, mm Hg:						
SBP, mean (SD)	151.9 (15.6)	138.9 (15.5)	<0.001	152.3 (14.9)	139.2 (15.4)	<0.001
DBP, mean (SD)	85.2 (11.1)	83.2 (9.3)	<0.001	83.1 (10.5)	82.5 (9.5)	0.09
Treated at baseline, mm Hg:						
SBP, mean (SD)	151.5 (15.6)	136.6 (14.9)	<0.001	151.9 (14.8)	137.6 (15.0)	<0.001
DBP, mean (SD)	85.0 (11.1)	82.2 (9.2)	<0.001	83.0 (10.4)	81.8 (9.5)	0.002
Untreated at baseline, mm Hg:						
SBP, mean (SD)	162.7 (13.3)	153.4 (10.9)	<0.001	162.3 (12.7)	152.1 (11.1)	<0.001
DBP, mean (SD)	89.6 (11.1)	88.9 (8.3)	0.69	85.6 (11.5)	87.6 (7.9)	0.14
Current cigarette smoker, *n* (%)	185 (19.9)	200 (27.8)	<0.001	243 (19.1)	336 (20.6)	0.32
Type 2 diabetes, *n* (%)	435 (46.8)	227 (31.6)	<0.001	531 (41.7)	458 (28.0)	<0.001
LVH by electrocardiogram, *n* (%)	273 (29.4)	149 (20.7)	<0.001	178 (14.0)	212 (13.0)	0.43
Body mass index, mean (SD), kg/m^2^	31.3 (8.3)	29.5 (6.4)	<0.001	29.9 (5.7)	28.9 (5.3)	<0.001
Fasting glucose, mean (SD), mg/dL	132.8 (62.5)	120.4 (60.8)	<0.001	124.8 (50.8)	114.8 (48.1)	<0.001
Total cholesterol, mean (SD), mg/dL	217.1 (45.4)	216.0 (44.7)	0.64	213.6 (43.9)	215.0 (41.7)	0.38
HDL cholesterol, mean (SD), mg/dL	50.6 (15.9)	52.8 (15.8)	0.008	42.9 (13.7)	45.1 (14.0)	<0.001
Fasting triglycerides, mean (SD), mg/dL	130.0 (75.9)	125.4 (91.9)	0.33	196.8 (142.9)	177.7 (126.0)	<0.001
Estimated glomerular filtration rate (eGFR)	80.3 (21.3)	86.2 (19.6)	<0.001	73.9 (18.3)	75.8 (16.5)	0.004
Follow-up blood pressure, mm Hg:						
SBP at 1 year, mean (SD)	151.4 (18.4)	129.1 (13.5)	<0.001	147.2 (17.7)	128.1 (12.2)	<0.001
DBP at 1 year, mean (SD)	83.8 (11.3)	78.1 (8.5)	<0.001	79.0 (10.4)	76.5 (8.6)	<0.001
SBP at 2 years, mean (SD)	149.6 (18.8)	127.9 (13.0)	<0.001	146.4 (17.2)	126.3 (11.9)	<0.001
DBP at 2 years, mean (SD)	81.4 (12.5)	77.1 (8.6)	<0.001	78.0 (10.8)	75.5 (7.9)	<0.001
SBP at 3 years, mean (SD)	152.3 (18.3)	117.2 (6.5)	<0.001	149.2 (16.9)	117.9 (6.3)	<0.001
DBP at 3 years, mean (SD)	81.1 (12.3)	73.1 (6.9)	<0.001	77.2 (11.6)	72.0 (7.2)	<0.001
Number of HT meds at 3 years, mean (SD)	3.6 (0.72)	1 (per def.)	—	3.6 (0.69)	1 (per def.)	—
Number on diuretic at 3 years, *n* (%)	493 (53%)	366 (51%)	0.38	769 (60%)	695 (43%)	<0.001

SBP: systolic blood pressure, DBP: diastolic blood pressure, HDL-C: HDL cholesterol, and LVH: left ventricular hypertrophy.

*Test of differences between case/control groups: ANOVA for continuous variables and chi-square for categorical variables.

**Participants were randomized to treatment in a ratio of 1 : 1 : 1 : 1.7 for amlodipine, lisinopril, doxazosin, and chlorthalidone, respectively.

**Table 3:** Association of clinical outcomes with treatment resistant hypertension.

Clinical outcomes	African American						White					
	Number of events		Event rates per 1000 person-years		Odds ratio (*P* value) for cases versus controls		Number of events		Event rates per 1000 person-years		Odds ratio (*P* value) for cases versus controls	
	TRH cases (*n* = 929)	Controls (*n* = 719)	TRH cases	Controls	Unadjusted	Adjusted*	TRH cases (*n* = 1274)	Controls (*n* = 1635)	TRH cases	Controls	Unadjusted	Adjusted*
CHD (fatal CHD or nonfatal MI)	82	25	18.0	6.9	2.66 (<0.001)	3.44 (<0.001)	159	75	26.2	9.4	2.80 (<0.001)	2.40 (<0.001)
Stroke	43	22	9.3	6.1	1.55 (0.10)	1.15 (0.66)	64	27	10.3	3.4	3.04 (<0.001)	2.19 (0.003)
Heart failure	104	22	23.2	6.0	3.86 (<0.001)	2.69 (<0.001)	184	50	30.7	6.2	5.03 (<0.001)	3.98 (<0.001)
End-stage renal disease	28	0	6.0	0	23.1 (0.002)**	10.9 (0.03)**	20	4	3.2	0.5	6.33 (0.001)	3.89 (0.02)
All-cause death	103	51	20.7	13.5	1.48 (0.02)	1.66 (0.01)	127	88	19.1	10.6	1.69 (<0.001)	1.35 (0.06)
Combined CHD	142	50	32.5	13.9	2.36 (<0.001)	2.84 (<0.001)	307	175	54.6	22.6	2.44 (<0.001)	2.22 (<0.001)
Combined CVD	277	110	69.4	32.0	2.18 (<0.001)	2.11 (<0.001)	510	282	100.7	38.0	2.70 (<0.001)	2.42 (<0.001)

*Adjusted for age, sex, randomized treatment group, baseline values of BMI, HDL, eGFR, SBP, and DBP, smoking status, diabetes status and history of LVH; **added 1 event into each case and control group to force estimate.

**Table 4:** Significant gene associations with treatment resistant hypertension.

Variant name	Rs number	Geno-type	African American				White			
			TRH cases	Controls	Odds ratio (95% CI)*	*P* value*	TRH cases	Controls	Odds ratio (95% CI)*	*P* value*
		TT	640 (70.6)	463 (66.5)			228 (18.2)	380 (23.5)		
AGTM235T	699	TM	237 (26.1)	203 (29.2)	0.83 (0.67–1.03)	0.10	597 (47.5)	801 (49.6)	1.27 (1.12–1.44)	0.0001
		MM	30 (3.3)	30 (4.3)			431 (34.3)	434 (26.9)		

		AA	658 (72.0)	496 (69.9)			226 (18.1)	401 (24.7)		
AGT-6G	5051	AG	231 (25.3)	187 (26.3)	0.90 (0.72–1.12)	0.35	597 (47.9)	806 (49.7)	1.36 (1.20–1.53)	<0.0001
		GG	25 (2.7)	27 (3.8)			424 (34.0)	414 (25.5)		

*Odds of being a case versus control for each copy of the minor allele (additive genetic model) after adjustment for age, sex, randomized treatment group, baseline values of BMI, HDL, eGFR, SBP, and DBP, smoking status, diabetes status, and history of LVH.

**Table 5:** Gene associations with treatment resistant hypertension that are suggestive for both AA and white participants.

Variant name	Rs number	Geno-type	African American				White			
			TRH cases	Controls	Odds ratio (95% CI)*	*P* value*	TRH cases	Controls	Odds ratio (95% CI)*	*P* value*
		CC	717 (78.8)	508 (72.7)			564 (44.8)	662 (40.9)		
MTHFR	1801133	CT	178 (19.6)	175 (25.0)	0.67 (0.52–0.86)	0.002	555 (44.1)	751 (46.4)	0.88 (0.77–0.99)	0.04
		TT	15 (1.7)	16 (2.3)			139 (11.1)	207 (12.8)		

		AA	750 (82.3)	553 (79.1)			345 (27.6)	502 (31.2)		
MMP3 5A/6A	3025058	*A	153 (16.8)	133 (19.0)	0.75 (0.57–0.98)	0.04	601 (48.1)	778 (48.4)	1.14 (1.01–1.29)	0.03
		**	8 (0.9)	13 (1.9)			304 (24.3)	327 (20.4)		

		GG	842 (92.7)	666 (95.0)			1115 (88.7)	1470 (91.0)		
TNF	361525	GA	63 (6.9)	34 (4.9)	1.87 (1.13–3.11)	0.02	139 (11.1)	143 (8.9)	1.44 (1.10–1.90)	0.009
		AA	3 (0.3)	1 (0.1)			3 (0.2)	2 (0.1)		

*Odds of being a case versus control for each copy of the minor allele (additive genetic model) after adjustment for age, sex, randomized treatment group, baseline values of BMI, HDL, eGFR, SBP, and DBP, smoking status, diabetes status, and history of LVH.
